# Treatment strategies after acute exacerbations of chronic obstructive pulmonary disease: Impact on mortality

**DOI:** 10.1371/journal.pone.0208847

**Published:** 2018-12-14

**Authors:** Fernando Casas-Mendez, Maria Jose Abadías, Oriol Yuguero, Ignasi Bardés, Ferran Barbé, Jordi de Batlle

**Affiliations:** 1 Group of Translational Research in Respiratory Medicine, Hospital Universitari Arnau de Vilanova de Lleida,IRBLleida, Universitat de Lleida, Lleida, Catalonia, Spain; 2 Emergency Department, Hospital Universitari Arnau de Vilanova de Lleida, Catalonia, Spain; 3 Emergency Department, Hospital Universitari de Bellvitge, Barcelona, Catalonia, Spain; 4 Centro de Investigación Biomédica en Red de Enfermedades Respiratorias (CIBERES), Madrid, Spain; Ospedale S. Corona, ITALY

## Abstract

**Introduction:**

Acute exacerbation of chronic obstructive pulmonary disease (AECOPD) is a common reason for presentation to emergency departments (ED), but the management of these episodes is often heterogeneous regardless of their potential impact on short-term adverse outcomes.

**Methods:**

This was a longitudinal, retrospective study of all patients >40 years old admitted to the ED of two Spanish teaching hospitals for an AECOPD between January 1^st^ and May 31^st^, 2016. All data were collected from electronic medical records. The primary outcomes were patient treatment at discharge and 90-day mortality. Logistic regression was used to model the determinants of 90-day mortality.

**Results:**

Of the 465 included patients, 56% were prescribed a 3-drug combination at hospital discharge, 22% a 2-drug combination, 19% a single drug, and 4% other or no treatment. Approximately 8% of patients died within 90 days after an AECOPD. Multivariate logistic models revealed that having more than 2 severe exacerbations within the last 12 months (OR (95% CI): 15.12 (4.22–54.22)) and being prescribed a single drug at discharge (OR (95% CI): 7.23 (2.44–21.38)) were the main determinants of 90-day mortality after an AECOPD.

**Conclusions:**

This study reflects the real-life heterogeneity in the pharmacological treatments prescribed after an ED admission for an AECOPD and suggests the potential impact of suboptimal inhaled treatment strategies on 90-day mortality rates.

## Introduction

An acute exacerbation of chronic obstructive pulmonary disease (COPD; AECOPD) is an episode of worsening of the patient’s respiratory symptoms (i.e., baseline dyspnea, cough, and/or sputum production) beyond the normal day-to-day variation that are sufficient to warrant a change in medication [1]. An AECOPD is the most relevant event affecting COPD mortality and, in fact, the frequency and severity of these episodes are the major modifiers the management and outcome of the disease [2]. COPD, particularly AECOPD, accounts for significant consumption of health care resources [3]. In Spain, AECOPD accounts for 1%-2% of all emergency department (ED) visits, and up to 10% of all medical admissions are related to COPD [4]; similar data have been reported elsewhere [5]. Moreover, it is estimated that one out of ten patients suffering from an AECOPD requiring in-hospital management die during the following 90 days [6]. Thus, AECOPD can be considered a major public health issue.

International guidelines support the use of inhaled bronchodilators as the cornerstone of management of COPD, and it is well known how these medications contribute to improving patient symptoms and quality of life [7]. Moreover, there is enough evidence that supports the use of inhaled bronchodilators in the prevention of disease progression and relapsing exacerbations [8]. However, unlike many other factors that have been associated with mortality [9], the influence of maintenance inhaled treatment on short-term prognosis after an acute exacerbation has not been elucidated yet. In this sense, understanding whether a particular bronchodilator or inhaled glucocorticoid treatment, used either alone or in combination, after an AECOPD can influence mortality may be very relevant not only because of the potential effects of these medications at the respiratory level but also because of their effects on the inflammatory response and their potential to reduce the risk of death due to cardiovascular causes [10]. In this scenario, we conducted a retrospective longitudinal study to assess clinical predictors of short-term adverse outcomes, including mortality, of different strategies of maintenance treatment prescribed at hospital discharge after an ED visit because of an AECOPD.

## Methods

### Study design and subjects

This was a longitudinal, retrospective study of the first 500 patients >40 years old admitted to the EDs of two Spanish hospitals because of an AECOPD (primary cause according to usual practice in the participating EDs) from January 1^st^ to May 31^st^, 2016. The participating centers were Arnau de Vilanova University Hospital (HUAV), in Lleida, and Bellvitge University Hospital (HUB), in Barcelona; both of them are regional reference hospitals. To be included in the current analyses, all patients had to have spirometric confirmation of COPD, according to international guidelines, or a previous diagnosis of COPD with specific treatment and follow-up with a primary care (PC) or secondary care (SC) physician. In the latter case, inclusion in the study required the confirmation of the case by a senior physician based on the information available in the electronic medical records. This confirmation was performed at the time of data collection, thus months after the ED admission.

Patients were excluded if they had any other identifiable cause of the worsening of their symptoms (pneumonia, pneumothorax, decompensated or unknown arrhythmia, ischemic heart disease, pulmonary thromboembolism or left heart failure); if they required invasive mechanical ventilation at the time of the first medical visit (as these patients are directly referred to the intensive care unit); or if they received ambulatory follow-up in a palliative care unit. Patients with more than one exacerbation requiring an ED visit during the study period were considered only once.

#### Clinical data and measurements

Based on the data available in the electronic medical records of each patient, the following variables were collected: sociodemographic and lifestyle data (age, sex, smoking status and alcohol consumption); comorbidities (age-modified Charlson index [11]); baseline characteristics of COPD (degree of obstruction, basal dyspnea, history of exacerbations during the last 12 months, “clinical phenotype” according to the Spanish national guidelines [12], and Global Initiative for Chronic Obstructive Lung Disease (GOLD) 2017 ABCD classification [7]); pharmacological treatment before and after ED admission; overall characteristics of the AECOPD in the ED (amount of time since the onset of symptoms, Anthonisen criteria, vital signs, pulse oxygen saturation, fraction of inspired oxygen, and any complementary tests); and variables related to the hospital admission (length of stay and in-hospital mortality). Any stay longer than 24 h in any hospital ward, unit or ED was considered a hospital admission. Finally, all participants were retrospectively followed for 90 days after discharge from the hospital or ED and the following information was collected from the primary care electronic medical records (ECAP): time to first PC assessment after the AECOPD; number of readmissions at 90 days; and mortality at 90 days.

The following pharmacological treatments for COPD were considered: short-acting beta agonists (SABA); long-acting beta agonists (LABA); short-acting muscarinic antagonists (SAMA); long-acting muscarinic antagonists (LAMA); inhaled corticosteroids (ICS); and other drugs (inhaled antibiotics, oral antibiotics, roflumilast, theophylline and n-acetylcysteine). However, the use of short-acting bronchodilators was ignored when these medications were prescribed as rescue or relief drugs only, and not as part of maintenance treatment. Pharmacological treatments were considered both individually and grouped as 3-drug, 2-drug or single-drug treatments, this reflecting treatment intensity. The 3-drug combinations included: LAMA + LABA + ICS; LAMA + SABA + ICS; and, LABA + SAMA + ICS. The 2-drug combinations included: LAMA + LABA; LAMA + SABA; LAMA + ICS; LABA + SAMA; LABA + ICS; and, SAMA or SABA + ICS. When considering drug combinations, the combination of LAMA + SAMA was considered as LAMA alone; the combination of LABA + SABA was considered as LABA alone; and, inhaled antibiotics, oral antibiotics, roflumilast, theophylline and n-acetylcysteine were grouped into the “other treatment” category and were not considered in the 3-drug, 2-drug or single-drug categories.

All data were collected by medical research staff and entered into an anonymized database. The ethics committee of the coordinator center (HUAV) approved the study (approval number 10/2015).

#### Statistical analysis

Baseline characteristics of included patients were described using the mean (standard deviation [SD]) or median (P25-P75) for continuous variables and absolute numbers and percentages for categorical variables. Bivariate analysis according to survival status at 90 days were performed using the Chi-square test, Student’s *t*-test, or the Mann-Whitney U test, depending on the characteristics of each variable. Logistic regression was used to model the determinants of 90-day mortality. A multivariate model including all variables that were statistically significant in the univariate models as well as age, gender, tobacco use and whether the patient required hospital admission was constructed. Additionally, stratified analyses according to gender and center were performed. All comparisons were bilateral, and a value of p<0.05 was considered statistically significant. All analyses were performed using Stata 12.1 software (StataCorp, College Station, TX, USA).

## Results

### Patient characteristics

The first 500 patients who presented with an AECOPD from January 1^st^ to May 31^st^, 2016, were considered for this analysis; 250 patients at the ED of HUAV and 250 at the ED of HUB were evaluated. Thirty-five patients were excluded (12 because they had spirometric values incompatible with the diagnosis of COPD, and 23 were excluded because they had a non-COPD diagnosis as the predominant cause of respiratory disease). Therefore, the study consisted of a sample of 465 patients: 217 (47%) from HUAV and 248 (53%) from HUB. Table 1 shows the main characteristics of the study population. Briefly, included patients had a mean (SD) age of 75 (11) years with an average modified Charlson index of 6.5 (2.1); spirometry was available in 81.5% of patients, with an average forced expiratory volume in the 1^st^ second (FEV_1_) of 50.5% (20.3%) of the predicted value; persistent dyspnea, which was defined as a score of 2 or more on the modified Medical Research Council (mMRC) dyspnea scale, was present in 80% of patients; 69% of patients were graded as C-D according to the GOLD 2017 ABCD classification; and approximately 58% of patients required hospital admission. No relevant differences in baseline characteristics and patient outcomes were found between the two hospitals.

**Table 1:** Main characteristics of the study population.

	n*	n (%)/mean (SD)/P50 (P25-P75)
**Sociodemographic variables**	** **	** **
Age, years	465	75.2 (11.2)
Sex	465	
Men		372 (80%)
Women		93 (20%)
Center	465	
Lleida		217 (47%)
Bellvitge		248 (53%)
**Lifestyle and comorbidities**	** **	** **
Tobacco use:	450	
Never		64 (14%)
Current		95 (21%)
Former		291 (65%)
Alcohol, units of alcohol/day	385	0 (0–2)
Age-modified Charlson index	464	6.5 (2.1)
Vaccination: Influenza	465	424 (91%)
Vaccination: PNC23	465	319 (69%)
Vaccination: Prevenar 13	465	57 (12%)
**Baseline COPD variables**	** **	** **
Clinical phenotype	449	
Non-exacerbator		52 (12%)
Exacerbator emphysema		112 (25%)
Exacerbator chronic bronchitis		211 (47%)
Asthma-COPD overlap		74 (16%)
GOLD 2017 ABCD classification	438	
A		123 (28%)
B		11 (3%)
C		225 (51%)
D		79 (18%)
Bronchiectasis	442	122 (28%)
Dyspnea, mMRC scale	459	
0/1		89 (20%)
2		276 (60%)
3		84 (18%)
4		10 (2%)
FVC, % predicted	377	66.8 (21.6)
FEV_1_, % predicted	378	50.5 (20.3)
FEV_1_/FVC, %	379	55.6 (14.8)
Mild/moderate exacerbations in the last 12 months	458	2 (1–3)
Severe exacerbations in the last 12 months	440	0 (0–1)
**Case evaluation**	** **	** **
Length of stay in the ED, h	465	9.4 (6.0)
Required hospital admission	465	270 (58%)
Ward/department during hospitalization	270	
Unspecified ward		63 (23%)
Respiratory department		60 (22%)
Internal medicine		110 (41%)
Geriatrics		35 (13%)
Home hospitalization		2 (1%)
Length of stay in the hospital, days	270	6.9 (4.7)
AECOPD outcome	465	
Deceased in the ED		6 (1%)
Deceased during hospitalization		16 (4%)
Discharged		443 (95%)
Rehospitalization at 90 days	440	108 (25%)
Deceased at 90 days	443	33 (8%)

PNC23: 23-valent pneumococcal polysaccharide vaccine; mMRC: modified Medical Research Council; FVC: forced vital capacity; FEV_1_: forced expiratory volume in the 1^st^ second; ED: emergency department; and AECOPD: acute exacerbation of chronic obstructive pulmonary disease.

* Subjects with available information (no missing information) from a total of 465 subjects in the study.

Table 2 shows the pharmacological treatments before ED admission and at discharge from the ED or at discharge from the hospital, if admission was required. Briefly, approximately 56% of patients were prescribed a 3-drug combination at hospital discharge, 22% a 2-drug combination, 19% a single drug, and 4% other or no treatment. Approximately 74% of patients were discharged without any changes in their baseline treatment, and only 20% of patients had an increase in the intensity of their treatment with the addition of inhaled drugs from a different class of medications.

**Table 2:** Pharmacological treatment before ED admission and at hospital discharge.

	Previous treatment	Treatment at discharge
**Treatment combinations**	** **	** **
LAMA + LABA + ICS	93 (20%)	80 (18%)
LAMA + SABA + ICS	0 (0%)	1 (0%)
LABA + SAMA + ICS	139 (30%)	166 (38%)
LAMA + LABA	31 (7%)	39 (9%)
LAMA + SABA	8 (2%)	8 (2%)
LAMA + ICS	6 (1%)	4 (1%)
LABA + SAMA	7 (2%)	6 (1%)
LABA + ICS	39 (8%)	29 (7%)
SAMA or SABA + ICS	13 (3%)	12 (3%)
LAMA	26 (6%)	18 (4%)
LABA	6 (1%)	2 (1%)
SABA or SAMA	46 (10%)	59 (13%)
ICS	3 (1%)	3 (1%)
Other*	0 (0%)	8 (2%)
Nothing	48 (10%)	8 (2%)
**Categorized treatment combinations**		** **
3-drug combination	232 (50%)	247 (56%)
2-drug combination	104 (22%)	98 (22%)
Single drug	81 (18%)	82 (19%)
Other* or nothing	48 (10%)	16 (4%)

LAMA: long-acting muscarinic antagonists; LABA: long-acting beta agonists; ICS: inhaled corticosteroids; SABA: short-acting beta agonists; and SAMA: short-acting muscarinic antagonists.

* Other drugs: inhaled antibiotics, oral antibiotics, roflumilast, theophylline or n-acetylcysteine.

The combination of LAMA + SAMA was considered LAMA alone; and the combination of LABA + SABA was considered LABA alone. The use of short-acting bronchodilators was ignored except when these medications were prescribed as part of maintenance treatment and not only as rescue or relief drugs.

Table 3 describes the relationship between the treatment at discharge and survival status at 90 days after discharge. The lowest mortality rates (5%) were found among patients receiving a 3-drug combination, while the highest (15%) were found among patients receiving a single-drug treatment. The main determinants of mortality during the 90 days following an AECOPD are shown in Table 4. Briefly, crude analyses showed that treatment, age, past severe exacerbations and age-modified Charlson index were positively associated with an increased mortality risk. Adjusted analyses confirmed that treatment and previous severe exacerbations were the main determinants of 90-day mortality. The number of combined treatments was significantly associated with a lower 90-day mortality (p-for-trend = 0.002). The effect of variables such as age or a requirement for hospital admission during an AECOPD did not reach statistical significance in the adjusted models. Finally, sensitivity analyses excluding patients without a spirometric confirmation of COPD showed similar results (S1 Additional Results).

**Table 3:** Pharmacological treatment at hospital discharge according to survival status at 90-days after an AECOPD.

	Alive	Deceased	
	n (%)	n (%)	p-value*
Categorized treatment combinations			**0.027**
3-drug combination	235 (95%)	12 (5%)	
2-drug combination	91 (93%)	7 (7%)	
Single drug	70 (85%)	12 (15%)	
Other** or nothing	14 (88%)	2 (13%)	

* Chi-square test.

** Other drugs: inhaled antibiotics, oral antibiotics, roflumilast, theophylline or n-acetylcysteine.

The combination of LAMA + SAMA was considered LAMA alone; and the combination of LABA + SABA was considered LABA alone. The use of short-acting bronchodilators was ignored except when these medications were prescribed as part of maintenance treatment and not only as rescue or relief drugs.

LAMA: long-acting muscarinic antagonists; LABA: long-acting beta agonists; SABA: short-acting beta agonists; and SAMA: short-acting muscarinic antagonists.

**Table 4:** Main determinants of mortality during the 90 days following an AECOPD.

	Crude *		Adjusted **	
	OR	95% CI	OR	95% CI
Categorized treatment combinations:				
3-drug combination	ref		ref	
2-drug combination	1.54	0.54–4.37	1.81	0.57–5.73
Single drug	**4.43**	**1.83–10.77**	**7.23**	**2.44–21.38**
Other*** or nothing	3.35	0.67–16.91	3.31	0.49–22.31
Sex: woman	0.66	0.22–1.95	0.89	0.19–4.16
Age	**1.04**	**1.00–1.08**	1.04	0.98–1.10
Center: Bellvitge	0.71	0.33–1.49	0.65	0.27–1.59
Tobacco use:				
Never	ref		ref	
Current	1.13	0.26–4.94	1.05	0.15–7.31
Former	1.59	0.46–5.52	0.86	0.16–4.61
Mild/moderate exacerbations in the last 12 months				
0	ref		ref	
1	2.37	0.51–11.01	2.09	0.39–11.09
2	0.78	0.13–4.81	0.63	0.09–4.62
3+	2.27	0.49–10.44	2.01	0.35–11.59
Severe exacerbations in the last 12 months				
0	ref		ref	
1	1.34	0.48–3.74	1.40	0.45–4.34
2	2.04	0.61–6.71	2.39	0.61–9.33
3+	**9.16**	**3.40–24.73**	**15.12**	**4.22–54.22**
Requires hospital admission	1.44	0.66–3.16	1.58	0.59–4.22
Age-modified Charlson index	**1.27**	**1.07–1.50**	1.08	0.87–1.36

* A model for each variable (n = 405 in all models).

** A single model including all variables (n = 405).

*** Other drugs: inhaled antibiotics, oral antibiotics, roflumilast, theophylline or n-acetylcysteine.

The combination of LAMA + SAMA was considered LAMA alone; and the combination of LABA + SABA was considered LABA alone. The use of Short-acting bronchodilators was ignored except when these medications were prescribed as part of maintenance treatment and not only as rescue or relief drugs.

AECOPD: acute exacerbation of chronic obstructive pulmonary disease; LAMA: long-acting muscarinic antagonists; LABA: long-acting beta agonists; SABA: short-acting beta agonists; and SAMA: short-acting muscarinic antagonists.

## Discussion

This study highlights the heterogeneity in the pharmacological treatments prescribed after an ED admission because of an AECOPD and the relationship of these different treatments to 90-day mortality. As expected, patients with a previous history of repeated severe COPD exacerbations showed an increased risk of short-term mortality after an AECOPD. However, in this real-world population, the pharmacological strategy of inhaled treatments prescribed at discharge from the hospital or the ED was found to have a significant impact on 90-day mortality rates. Specifically, low-intensity treatments, defined as the use of a single maintenance drug, were associated with higher mortality rates than 3-drug combination treatments, regardless of the severity of the AECOPD. This study also revealed that the global therapeutic approach for high-risk patients with COPD after presenting to the hospital with an AECOPD did not always correspond to the recommendations based on the best available scientific evidence.

There is currently enough evidence that supports the use of pharmacological therapy for COPD to reduce symptoms, reduce the frequency and severity of exacerbations and increase exercise tolerance and overall health status [13,14]. However, to date, few studies have assessed whether bronchodilators or ICS can significantly affect mortality or improve lung function or whether these effects are a result of these drugs used separately or used in combination in a single device [15–18]. A retrospective study of 4,263 patients showed a 42% reduction in the risk of death or readmission in the year after the first severe AECOPD in patients who received treatment with LABA/ICS compared to those who received less intensive therapy (ICS, LABA or SABA) [19]. Gudmunson et al. showed that both LABA and the LABA/ICS combination were associated with a decrease in the risk of death after a hospital admission due to an AECOPD [20]. Finally, a retrospective study of 1,185 patients by Sarc et al. found that long-term mortality after an AECOPD might be related to pharmacological treatment, since non-survivors were prescribed LABA, ICS and tiotropium less frequently and were prescribed theophylline more frequently [21].

The potential effects of bronchodilators and ICS as well as combinations of these medications on mortality can be explained by several mechanisms: first, inhaled treatment has been demonstrated to be a preventive measure against subsequent exacerbations due to its effects on hyperinflation and mechanical stress, decreased mucus production and enhanced mucociliary clearance [22]; and second, potential anti-inflammatory properties can have a positive impact on endothelial function and blood pressure, similar to how lung volume reduction surgery in patients with severe COPD reduces cardiovascular mortality [23]. However, our findings may be the result of an inadequate risk assessment by the attending physician, leading to suboptimal prescription of inhaled treatments. This possibility is supported by a recent big data analysis performed in Spain that proposed that suboptimal treatment, even in patients with severe COPD, may persist due to an underestimation of the significance of the disease or an ignorance about the indications and benefits of different pharmacological treatments [24]. Finally, it can be argued that patients who do not require hospitalization and thus are discharged directly from ED, may be prescribed suboptimal treatments due to the assumption that choosing the most appropriate medications and providing training with different inhalation devices are the responsibilities of the PC physician. Nevertheless, this argument does not apply to patients with severe exacerbations requiring hospitalization, who are at elevated risk of adverse events after discharge from the hospital.

Previous studies investigating risk factors for rehospitalization and death in patients with severe AECOPD reported similar short-term mortality rates [25–27]. The main determinant of 90-day mortality in our study was severe exacerbations during the previous 12 months. It is well known that previous hospitalizations for an AECOPD are independent predictors of respiratory and all-cause mortality [26,28,29], and each new hospitalization significantly increases the mortality rate during the subsequent 3 months [30]. Our results are fully aligned with the previous literature and highlight the relevance of previous severe exacerbations as predictors of short-term mortality after an AECOPD.

The main strengths of this study are its real-life setting, which reflects the broad range of combined therapies prescribed that are not always in agreement with the current guidelines, and its multicenter design, which captures the combined heterogeneity of two different hospitals with different procedures. On the other hand, several limitations should be acknowledged: (i) The retrospective design implies a degree of missing data, as only the information successfully recorded in the ED, hospital and PC records was available. (ii) Up to 19% of patients did not have spirometric confirmation of COPD, which highlights deficiencies in the assessment and/or recording of key information of COPD patients. To be included in the current analysis, patients without spirometric confirmation of COPD had the diagnosis confirmed by a comprehensive review of their medical records, which included a COPD diagnosis with specific treatment and follow-up with a PC or SC physician, as successfully implemented in previous studies [9–13, 30]. (iii) No information on patients’ treatment compliance was available. However, the impact of misclassification that any noncompliant patient might have on the study results would necessarily cause regression to the null effect and would not influence any already statistically significant results. (iv) In order to describe the most realistic scenario, patients with asthma-COPD overlap were not excluded from the study, even if these patients might experience additional benefits from treatment with ICS.

In conclusion, this study reflects the real-life heterogeneity in the pharmacological treatments prescribed after an ED admission for an AECOPD and suggests the potential impact of suboptimal inhaled treatment strategies on 90-day mortality rates. Specifically, low-intensity treatment, defined as the use of a single maintenance drug, appeared to be associated with higher mortality rates than 3-drug combination treatments, regardless of patient severity. Moreover, we confirmed the key role of previous severe COPD exacerbations as determinants of 90-day mortality. Overall, we highlighted the importance of appropriate assessment and treatment of patients suffering from an AECOPD according to the most updated guidelines, specifically those regarding inhaled therapy.

## Supporting information

S1 Additional Results Main determinants of mortality during the 90 days following an AECOPD, excluding patients without a spirometric confirmation of COPD. (DOCX)
