# Supplementary material for: Treatment strategies after acute exacerbations of chronic obstructive pulmonary disease: Impact on mortality
Source: PLoS One. 2018 Dec 14;13(12):e0208847. doi: 10.1371/journal.pone.0208847 (PMC6294427; doi:10.1371/journal.pone.0208847)
Supplement: S1 Additional Results — (DOCX) [file pone.0208847.s001.docx]

**S1 – Additional results**

**Treatment Strategies after Acute Exacerbations of Chronic Obstructive Pulmonary Disease: Impact on Mortality**

Fernando Casas-Mendez; Maria Jose Abadias; Oriol Yuguero; Ignasi Bardés; Ferran Barbé; Jordi de Batlle.

**Table 1. Main determinants of mortality during the 90 days following an AECOPD, excluding patients without a spirometric confirmation of COPD.**

|  | OR* | 95% CI |
| --- | --- | --- |
| Categorized treatment combinations: |  |  |
| 3-drug combination | ref |  |
| 2-drug combination | 2.15 | 0.58 - 7.94 |
| Single drug | **11.20** | **3.09 - 40.59** |
| Other** or nothing | - | - |
| Sex: woman | 0.63 | 0.09 - 4.60 |
| Age | **1.08** | **1.01 - 1.16** |
| Center: Bellvitge | 1.90 | 0.63 - 5.73 |
| Tobacco use: |  |  |
| Never | ref |  |
| Current | 1.73 | 0.12 - 25.12 |
| Former | 0.70 | 0.06 - 8.57 |
| Mild/moderate exacerbations in the last 12 months |  |  |
| 0 | ref |  |
| 1 | 0.81 | 0.25 - 2.61 |
| 2 | 0.39 | 0.09 - 1.89 |
| 3+ | - | - |
| Severe exacerbations in the last 12 months |  |  |
| 0 | ref |  |
| 1 | 1.52 | 0.40 - 5.82 |
| 2 | 2.29 | 0.45 - 11.71 |
| 3+ | **19.11** | **4.18 - 87.27** |
| Requires hospital admission | 1.40 | 0.45 - 4.34 |
| Age-modified Charlson index | 0.96 | 0.72 - 1.27 |

* A single model including all variables (n = 300).

** Other drugs: inhaled antibiotics, oral antibiotics, roflumilast, theophylline or n-acetylcysteine.

The combination of LAMA + SAMA was considered LAMA alone; and the combination of LABA + SABA was considered LABA alone. The use of Short-acting bronchodilators was ignored except when these medications were prescribed as part of maintenance treatment and not only as rescue or relief drugs.

AECOPD: acute exacerbation of chronic obstructive pulmonary disease; LAMA: long-acting muscarinic antagonists; LABA: long-acting beta agonists; SABA: short-acting beta agonists; and SAMA: short-acting muscarinic antagonists.
